# Disparities in Tumor Microenvironment Between Primary and Metastatic Colorectal Cancer: Impact on Immune Infiltration and Survival

**DOI:** 10.3390/cancers18040566

**Published:** 2026-02-09

**Authors:** Ewelina Dziąg-Dudek, Aleksandra Semeniuk-Wojtaś, Magdalena Modzelewska, Arkadiusz Lubas, Letycja Róg, Barbara Górnicka, Rafał Stec

**Affiliations:** 1 Oncology Department, Medical University of Warsaw, 02-097 Warsaw, Poland; 2 Pathomorphology Department, Medical University of Warsaw, 02-097 Warsaw, Poland; 3 Department of Internal Medicine, Nephrology and Dialysis, Military Institute of Medicine—National Research Institute, 04-141 Warsaw, Poland

**Keywords:** colorectal cancer, tumor microenvironment, immune cells, CD8, CD68, PD-L1, metastases

## Abstract

Background/Objectives: In recent years, growing evidence that the tumor microenvironment (TME) plays crucial roles in the progression and treatment responses of various cancers has emerged. Unfortunately, we still do not fully understand the mechanisms through which the TME influences cancer development. Therefore, the aim of this study is to assess the impact of the TME on the clinical course of the disease, comparing primary and metastatic tumors. Materials and Methods: This retrospective study included 30 colorectal cancer patients for which tissue samples from primary and metastatic tumors were available for immunohistochemistry. A multiple Cox proportional hazards regression analysis was performed to characterize differences between the microenvironments of primary and metastatic tumors, as well as between lesions diagnosed at different times after resection. Results: Immune cell infiltration was higher in metastatic than primary tumors. Statistically significant differences were observed only in the central part of the tumor, while cell infiltration at the periphery had no prognostic significance. In the multivariate analysis, a positive correlation was revealed between the expression of Programmed Death-Ligand 1 (PD-L1) on primary tumor cells (TCs) and survival (HR: 5.43; 95% CI: 1.89–15.61; *p* = 0.0017). Conclusions: Primary and metastatic tumors differ regarding their tumor microenvironment. As such, the tumor immune status should be considered as a key factor when selecting a therapeutic strategy, as well as for post-treatment surveillance.

## Simple Summary

The tumor microenvironment (TME) is increasingly recognized as a key factor influencing the progression and response to treatment of cancers; however, its role in the evolution of the disease remains insufficiently understood. The aim of this retrospective study was to evaluate differences in the TME between primary and metastatic colorectal cancer tumors and to assess their potential impacts on the clinical course of the disease. Immunohistochemical analyses were performed on tissue samples obtained from primary and metastatic lesions, and immune cell infiltration characteristics were compared between tumor sites and across different stages of disease progression. Metastatic tumors exhibited more pronounced immune cell infiltration than primary lesions, with relevant differences observed predominantly within the central tumor region. These findings indicate that primary and metastatic tumors display distinct microenvironmental profiles. Assessment of the tumor immune landscape may therefore provide clinically relevant information and should be considered when planning therapeutic strategies and post-treatment surveillance in patients with colorectal cancer.

## 1. Introduction

Colorectal cancer (CRC) is one of the most common cancers in the world, being responsible for 10% of cancer cases in both sexes in 2022 [1]. Approximately 25% of patients have metastases at the time of diagnosis with colorectal cancer, and about 50% develop metastases during the disease. The five-year survival rate in CRC patients is about 60% [2]. The main method of treatment for patients with non-metastasized colorectal cancer is surgery, with adjuvant chemotherapy in justified cases. Unfortunately, despite treatment, the risk of recurrence is 30–50% and the cumulative risk of death due to colorectal cancer is 0.65% among men and 0.45% among women [3,4,5,6,7].While most relapses (more than 90%) are observed within 5 years after CRC treatment, there have also been reported cases of relapse after 10 years [4,8,9,10].

According to available data, the risk of recurrence is linked to the site and stage of the primary tumor, as well as factors such as age, gender, and tumor histopathology [11,12,13,14]. In addition, treatment-related factors—such as oxaliplatin dose intensity below 60%, capecitabine dose intensity below 80%, and fewer than four cycles of adjuvant therapy—have been associated with a higher risk of colorectal cancer recurrence [15,16].

Unfortunately, regardless of the abovementioned factors, CRC recurrences may also be diagnosed in stage I patients, with the recorded rate of CRC recurrence in the early stage being 2.4–4.6% [17,18,19,20]. Paik et al. reported a recurrence rate of 2.9% for TNM stage I colon cancer [21]. In a meta-analysis, Hwang et al. demonstrated that T2 stage, lymphatic vessel invasion, venous invasion, elevated CEA levels, rectal cancer, and left-sided colon cancer were identified as risk factors for recurrence in stage I colorectal cancer patients, closely overlapping with those observed in more advanced stages of CRC [22]. Keramati et al. demonstrated that T3 and T4 tumors were associated with significantly higher recurrence rates when compared with T ≤ 2 tumors (*p* = 0.009). However, even in the case of T3 and T4 tumors, not all patients experience disease recurrence [23].

In recent years, increasingly more research has been undertaken to explain the impacts of the tumor microenvironment (TME) on the course of the disease, as cancer cells are not fully autonomous. It is assumed that the extracellular matrix (ECM), lymphocytes B and T, natural killer cells (NK), dendritic cells (DC), macrophages, endothelial cells, and fibroblasts [9,24] may all influence the development of tumors and their responses to therapy [25,26,27,28,29]. In addition, cancer cells can evade the immune response initiated by immune system cells and, through secreted cytokines and direct interactions between cells, can stimulate leukocytes to secrete proteins that induce tumor development [9].

Previous studies have shown that primary colorectal cancers and their corresponding metastatic lesions may differ substantially regarding the immune tumor microenvironment. Angelova et al. demonstrated that metastatic lesions undergo immune-driven evolutionary changes across time and space, resulting in immune profiles that differ from those of primary tumors [30]. Van den Eynde et al. reported that both the composition and spatial organization of immune cells differ between primary colorectal tumors and metastatic sites, with consequences for prognosis and therapeutic responses [31]. Furthermore, Fridman et al. emphasized that qualitative differences in the immune context—rather than immune cell density, per se—are key determinants of tumor progression and treatment outcomes [32]. Despite these advances, direct comparative analyses of paired primary and metastatic tissues remain limited, particularly with respect to detailed immunohistochemical characterization, which motivated the present study.

The primary aim of this study is to evaluate the impacts of the tumor microenvironment on the clinical course of colorectal cancer by comparing tumors diagnosed at different time points following resection of the primary lesion, in addition to analyzing paired primary tumors and corresponding metastatic lesions obtained from the same patient. This paired study design minimizes interpatient variability and allows for biologically meaningful assessment of tumor microenvironmental changes during disease progression, representing an approach that is methodologically more robust than comparisons based on independent patient cohorts. Although such paired analyses remain relatively uncommon due to the limited availability of matched histopathological material from both primary and metastatic sites, the collected data may contribute to a better understanding of tumor biology and provide deeper insight into cancer cell–TME interactions and their involvement in tumor progression and distant metastasis formation, ultimately facilitating the development of new therapeutic strategies.

## 2. Materials and Methods

### 2.1. Materials

This retrospective study included 30 patients with colorectal cancer hospitalized at the University Clinical Center of the Medical University of Warsaw in 2010–2021. The patients included in the study had histopathologically confirmed synchronous or metachronous metastases. The patients’ clinical data and histopathological material of primary and metastatic tumors from the same patient were used. In patients with synchronous metastases, samples were obtained simultaneously during surgery while, in patients with metachronous metastases, samples were obtained at intervals during colectomy and metastasectomy or biopsy of the metastatic tumor(s).

Patients with no histopathological examination of metastatic tumors were excluded. The study was approved by the Bioethics Committee of the Medical University of Warsaw.

### 2.2. Immunohistochemistry

Tumor samples were evaluated by pathologists before immunohistochemical analysis. Slides were prepared from paraffin blocks and stained with hematoxylin and eosin (HE), following which representative areas were selected. Tumor zones with crush artifacts, necrosis, and regressive hyalinization were excluded. The degree of histological differentiation of the tumor was classified according to the WHO classification from 2010/2019, and the clinical stage of cancer was assessed based on the criteria of the fifth edition of the TNM (tumor, node, metastasis) classification developed by the International Society against Cancer (UICC, Union Internationale Contre le Cancer).

Next, immunohistochemical staining was performed on 4 µm-thick sections. Mouse monoclonal antibodies (DAKO) were used to assess the expression of the analyzed antigens CD4—clone 4B12 (Agilent Technologies/Dako, Glostrup, Denmark).; CD8—clone C8/144B (Agilent Technologies/Dako, Glostrup, Denmark); CD15—clone Carb-3 (Agilent Technologies/Dako, Glostrup, Denmark); CD56—clone 123C3 (Agilent Technologies/Dako, Glostrup, Denmark); CD68—clone KP1 (Agilent Technologies/Dako, Glostrup, Denmark); CD31—clone JC70A (Agilent Technologies/Dako, Glostrup, Denmark); PD-L1—clone 22C3 (Agilent Technologies/DakopharmDx, Glostrup, Denmark); αSMA—clone HHF35 (Agilent Technologies/Dako, Glostrup, Denmark), in addition to rabbit antibodies CD208—ab111090 (Abcam, Cambridge, UK). (Supplemmentary Materials). Immunohistochemical analyses were performed according to the manufacturer’s instructions, and slides were scanned using a Hamamatsu NanoZoomer 2.0 HT scanner (Hamamatsu, Japan).

CD4, CD8, CD15, CD56, CD68, CD31, CD208, αSMA, and PD-L1 antigens were analyzed in two samples from each patient: one from the primary tumor and another from a metastatic tumor. Additionally, the tumor center (CENTR) and invasive margin of the tumor (INV) were determined for each sample, and immune cell infiltration (as determined using the abovementioned tumor antigens)was examined in three random sites for each of these areas. The results are presented as the percentage of stained cells in relation to all cells in the examined area, as the number of cells per 1 mm^2^, and/or as an average value. Zones with necrotic or coagulative damage were excluded from the analyses.

Unfortunately, the CD15 antigen was only assessed in the invasive margin of the tumor as, in most cases, it was also stained on tumor cells, making it impossible to count the number of neutrophils. The same applied to CD208, which was present not only on dendritic cells but also tumor cells, making it impossible to completely distinguish and count dendritic cells following immunohistochemical staining, ultimately resulting in the abandonment of further CD208 antigen analysis.

CD31 expression was evaluated by quantifying the density of immunoreactive microvessels per 1 mm^2^ in samples obtained from both primary and metastatic tumors. PD-L1-positive cells were analyzed using a sequential assessment approach. Initially, representative tumor regions were identified at ×10 magnification, after which a comprehensive evaluation was performed at higher magnifications (×20 and ×40). PD-L1 expression was examined independently in tumor cells and immune cells, and the proportions of the tumor area containing PD-L1-positive tumor cells (TCs) and tumor-infiltrating immune cells (ICs) were calculated. Membranous staining was required to define PD-L1 positivity in TCs, while both membranous and cytoplasmic staining patterns were accepted for ICs. Evaluation of each specimen focused exclusively on the proportion of stained cells, regardless of staining intensity.

The immunohistochemical results were classified into three categories on a 1–3 scale, based on the percentage of PD-L1-positive cells: <1% (low), 1–5% (moderate), and >5% (high). Only intratumoral immune cells were included in the analysis. The staining intensities of antibodies against CD4, CD8, CD15, CD208, CD56, CD68, αSMA, and CD31 were quantified using the Qu Path software (version 0.2.3).

The analyzed antigens and their corresponding immune cell populations within the tumor microenvironment are summarized in Table 1.

### 2.3. Statistical Analysis

All statistical evaluations were performed using the Statistica software (Tibco Software Inc., Greenwood Village, CO, USA), version 13.3. Missing values were neither imputed nor otherwise corrected. A *p*-value below 0.05 was considered indicative of statistical significance. Results are reported as frequencies with percentages or as medians with corresponding extreme values. Associations between variables were examined through Spearman or Pearson correlation analyses, depending on data normality (as assessed via the Shapiro–Wilk test). Relationships between dichotomous and continuous variables were analyzed using the point-biserial correlation coefficient. The differences were tested using a *t*-test for independent or dependent variables, depending on the distribution’s normality; otherwise, a Mann–Whitney or Wilcoxon test was performed. Two-tailed power analysis was conducted for the estimated difference or correlation between variables and survival. The influences of immunohistochemically derived variables on recurrence-free survival (RFS) were investigated using univariable and multivariable Cox proportional hazards regression models. Optimal cutoff values distinguishing patient groups with different outcomes were identified via receiver operating characteristic (ROC) analysis. RFS was further analyzed using Kaplan–Meier survival curves, with stratification according to the selected variables, and group differences were evaluated using the F-Cox test.

The summary figures included in this manuscript were prepared with the assistance of ChatGPT (version 5.2) as a supportive tool for figure conceptualization and layout.

## 3. Results

### 3.1. Clinicopathological Characteristics of the Study Population

The analyzed group consisted of 30 patients. The overall median follow-up period was 49.6 (range: 2.9–109.6) months, and the median age was 65.35 (range: 45.1–87.9) years. The characteristics of the patient group are detailed in Table 2.

### 3.2. Immunohistochemical Characterization of Tumor Microenvironment Components

**Scheme 1:**
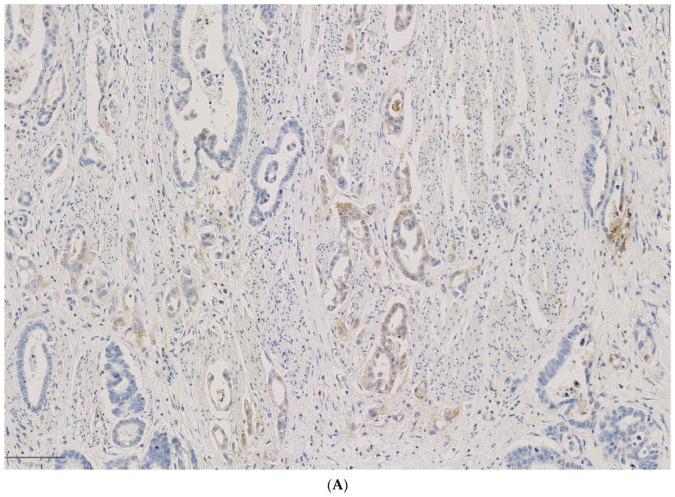

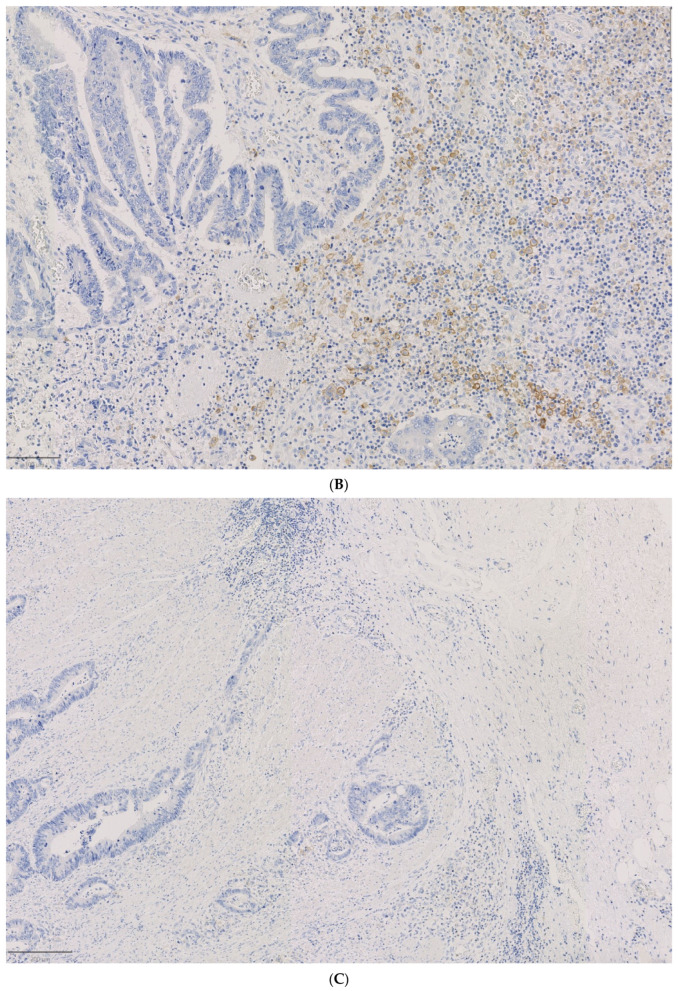

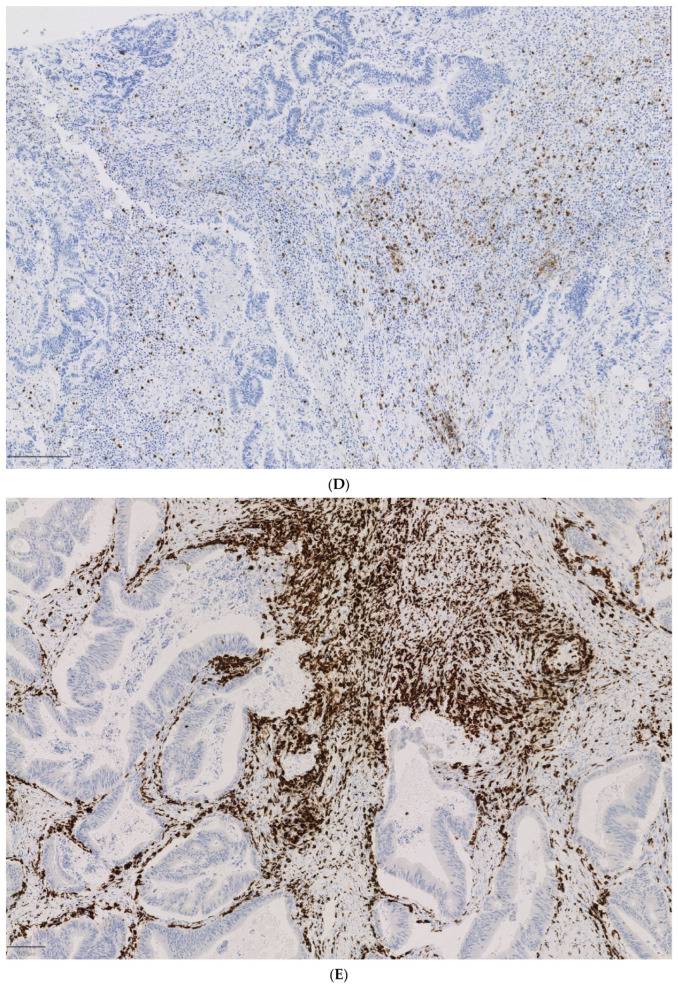
(×20): (**A**) high PD-L1 TC% expression; (**B**) high PD-L1 IC% expression; (**C**) low PD-L1 TC% expression; (**D**) CD8 tumor center density; (**E**) CD68 tumor center density.

Using immunohistochemistry, the levels of CD4, CD8, CD15, CD56, CD68, CD31, αSMA, and PD-L1 were assessed in colorectal cancer specimens and associated metastases, with the results summarized in Table 3a,b. Representative photographs are also presented (Scheme 1).

The analysis of immune cell density in the tumor revealed a higher level of infiltration at the invasive margin compared with the tumor center. Moreover, immune cell infiltration was greater in metastatic than primary tumors.

### 3.3. Correlations Between Tumor Microenvironment Components

The conducted analyses demonstrated correlations between overall survival (OS) and components of the tumor microenvironment (TME) in the primary tumor. Moreover, TME components were correlated with the presence of tumor budding, KRAS mutation, and the presence of perforation, as well as location and number of metastases and time of their occurrence (Table 4 and Table 5).

A correlation was found between pCD8 TC (tumor cells) or pPD-L1 TC and metastasis in the liver or pancreas. Moreover, the analyses revealed correlations between pCD56 TC or pCD15 INV (invasive margin of the tumor) and the number of metastases. The expression of pCD68 INV was correlated with the presence of tumor budding, and we also observed a correlation between pαSMA INV and tumor budding or the presence of angioinvasion. In addition, pCD8 INV correlated with the presence of perforation.

The frequency of pCD68 TC was correlated with the location of metastases, the time to occurrence of metastasis, the presence of KRAS mutation, and the presence of neuroinvasion.

The analyses carried out revealed a correlation between pCD4 TC and the presence of KRAS mutation.

The density of blood vessels in the invasive margin of the primary tumor (as indicated by pCD31 INV) was correlated with the presence of other tumors and the presence of perforations in contrast; meanwhile, the density of blood vessels in the center of the primary tumor (as indicated by pCD31 TC) was positively correlated with the presence of perforations.

In the analyzed metastatic samples, correlations were also found between TME components and the tumor location, the presence of neuroinvasion, and the time to occurrence of metastasis. Correlations between mCD8 TC or mCD68 TC and neuroinvasion were observed, as well as a correlations between the expression of mPD-L1 TC or mαSMA INV and the tumor being located on right or left side of the colon. The analyses carried out revealed that mCD15 INV was correlated with the time to the occurrence of metastasis. Furthermore, mCD68 TC was correlated with the presence of angioinvasion.

All correlations are detailed in Table 4 and Table 5.

**Figure 1:**
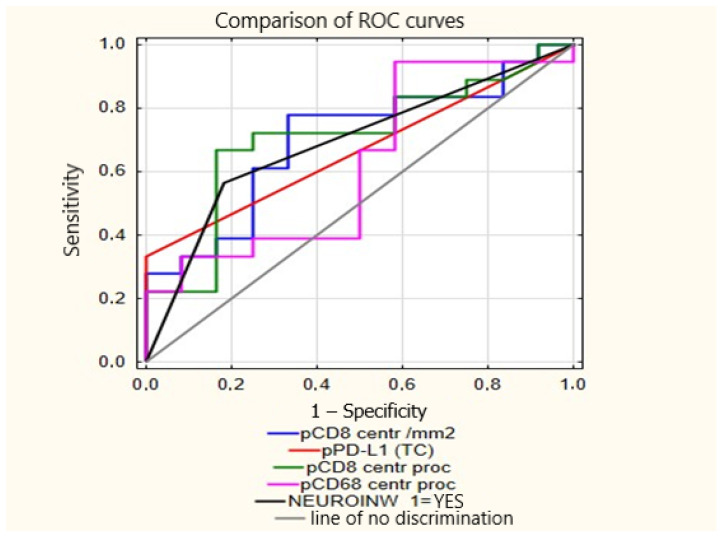
Comparison of ROC curves for the evaluated predictive markers.

**Figure 2:**
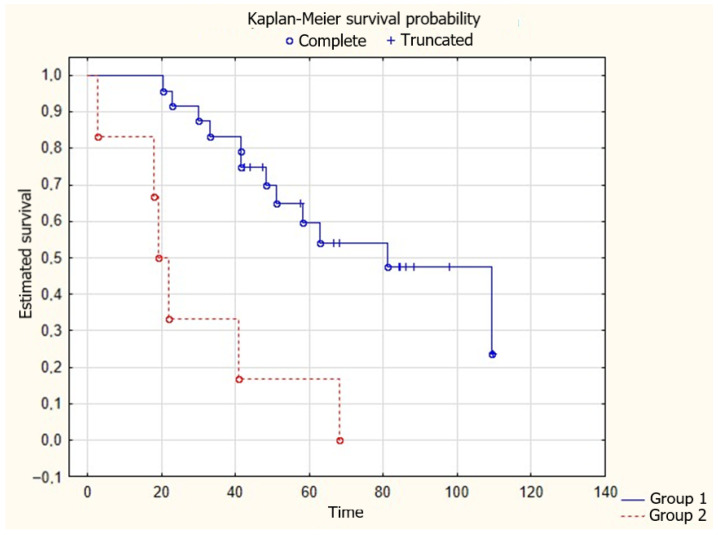
Kaplan–Meier survival curves according to PD-L1 expression on primary tumor cells. Group 1—low expression, <1% of tumor cells; group 2—moderate expression, 1–5% of tumor cells; F-Cox test: *p* = 0.00129; log-rank: *p* = 0.007, power 78.55%. Red and blue symbols means the same.

**Figure 3:**
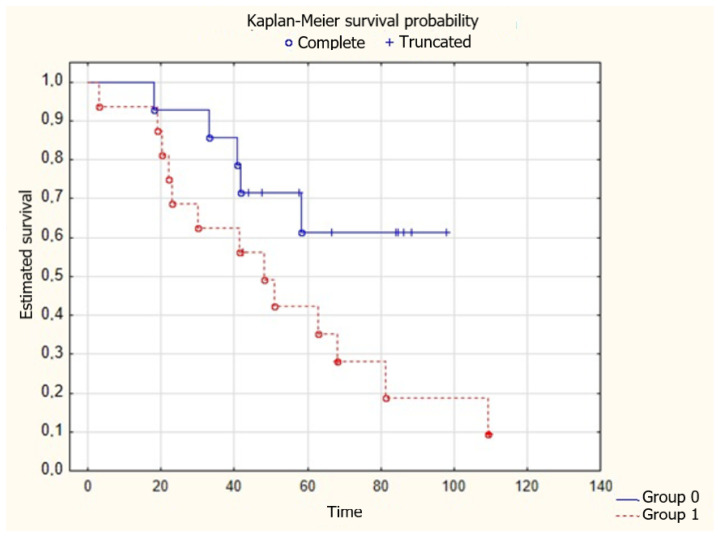
Kaplan–Meier survival curves according to CD8 expression on primary tumor cells in tumor center. Group 0—pCD8centr < 2% expression; F-Cox test: *p* = 0.00756; log-rank: *p* = 0.063, power 82.78%. Group 1—pCD8centr ≥ 2% expression. Red and blue symbols means the same.

**Figure 4:**
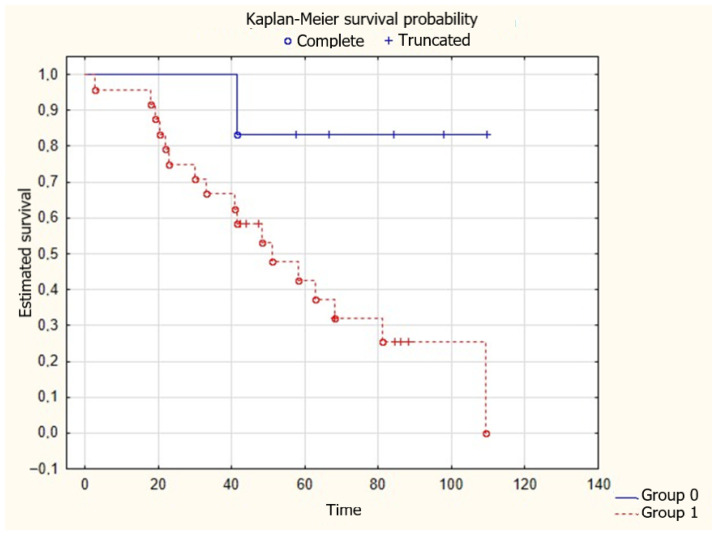
Kaplan–Meier survival curves according to CD68 expression on primary tumor cells in tumor center. Group 0—pCD68centr < 15.3% expression; F-Cox test: *p* = 0.00374; log-rank: *p* = 0.012, power 82.09%. Group 1—pCD68centr ≥ 15.3% expression. Red and blue symbols means the same.

**Figure 5:**
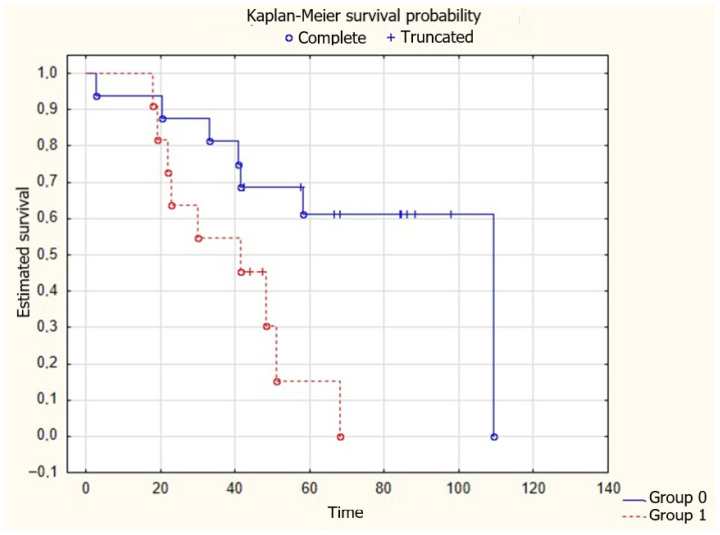
Kaplan–Meier survival curves according to presence of neuroinvasion in primary tumor. Group 0—No; Group 1—Yes; F-Cox test: *p* = 0.02004; log-rank: *p* = 0.018, power 68.82%.

In the univariable analysis, the expression of CD8, CD68, and PD-L1 on the center primary tumor cells (pTCs) and the presence of neuroinvasion were statistically significantly correlated with overall survival (*p* = 0.018, 0.039, 0.0008, and 0.016, respectively; see Table 6), (Figure 1, Figure 2, Figure 3, Figure 4 and Figure 5).

Additionally, in the multivariable analysis, a positive correlation was revealed between the expression of PD-L1 on primary tumor cells (TCs) and survival (HR: 5.43; 95% CI: 1.89–15.61; *p* = 0.0017; see Table 7).

In summary, significant differences were observed only in the primary tumor—specifically, in its center—whereas those relationships relating to the invasive margin were not substantial.

For variables found to be significantly associated with OS, a receiver operating characteristic (ROC) analysis was performed (Figure 1).

We additionally performed the Wilcoxon signed-rank test, which demonstrated a significant increase in the number of CD8+ cells in metastatic lesions compared with primary tumors, both in absolute count per mm^2^ (power of the difference test = 94.95%) and as a percentage of all cells within the respective invasive margin of the tumor. However, no significant differences were observed in CD8+ cell infiltration within the central tumor area (both percentage and density), indicating that the central compartment shows relative stability between primary and metastatic sites (Table 8).

Similarly, a statistically significant higher number of PD-L1-positive tumor-infiltrating immune cells was observed in metastases compared with the primary tumor (*p* = 0.03; see Table 8). For PD-L1 expression, no significant differences were found in tumor cells (*p* = 0.13), suggesting that PD-L1 status on immune cells differs between primary and metastatic tumors, but remains relatively constant on tumor cells.

In metastatic lesions, we found that a higher number of PD-L1-positive tumor-infiltrating immune cells was associated with a greater number of CD8+ tumor-infiltrating lymphocytes (TILs), both in absolute count per mm^2^ (power of the correlation test = 76.52%) and as a percentage of all cells within the respective invasive margin of the tumor. No such association was observed in primary tumors, nor between the number of CD8+ TILs and PD-L1-positive tumor cells (Table 9).

Furthermore, the Mann–Whitney U-test revealed a significant difference in the number of CD8+ cells within the invasive margin of metastatic lesions, depending on the level of PD-L1 expression on positive immune cells infiltrating the tumor (Table 10); in contrast, no substantial differences were detected in CD8+ infiltration within the central tumor region (*p* > 0.05).

## 4. Discussion

A cancer cell does not exist completely on its own as a single cell; it interacts with the entire microenvironment of the primary tumor and/or the microenvironment of the organ in which metastases form. The tumor microenvironment (TME) contributes to cancer cell proliferation, mutation acquisition, angiogenesis, activation of invasion and metastasis, reprogramming of energy metabolism, and evasion of immune destruction [45,46].

Distant metastases differ from the primary tumor at the molecular and genomic levels, with documented differences mainly including the composition of the TME [9,47,48], as well as changes in mutation expression between primary and metastatic lesions, such as differences in KRAS mutation demonstrated in patients with colorectal cancer (CRC) [49].

Cancer cells that settle in an organ where metastases develop are exposed to a new, hostile microenvironment with a distinct immune composition, which may render them susceptible to immune surveillance [50]. Moreover, the tumor microenvironment of secondary lesions differs depending on the organ in which they develop [51].

Wei et al. compared primary CRC tumor tissue and liver metastases, and noticed that PD-L1 expression and CD4+ lymphocyte infiltration density were higher in liver metastases than primary tumors in certain subgroups. In addition, in the analyzed samples, the expression of PD-L1—which can inhibit T cell activation and enable tumor immune evasion [52,53]—was positively correlated with CD4 and CD8 cell densities in liver metastases [54]. CD4+ T-lymphocytes can produce cytokines, recognize antigens presented by MHC class II molecules on antigen-presenting cells (APCs), and may either promote or inhibit tumor cell growth [55,56,57]. CD8+ tumor-infiltrating lymphocytes (TILs) contribute to tumor rejection by recognizing tumor-associated antigens presented by MHC class I molecules and directly killing target cells. Likewise, they secrete cytokines which enhance the cytotoxic function of TILs and promote a targeted antitumor immune response [58,59].

Similarly, Zhou et al. compared the densities of CD3+, CD8+, CD11b+, CD11c+, and CD33+ immune cell infiltrates in CRC primary tumor tissues and liver metastases.They found a greater abundance of immunosuppressive cells in metastatic lesions compared with primary tumors, and liver metastases were characterized by higher expression of CD33—a marker of myeloid-derived suppressive cells (MDSCs)—than the primary tumor. In contrast, CD8+ and CD3+ cells (which are universal T cell markers) showed higher expression in the primary tumor than in metastases [60]. These identified dependencies may partially account for the reduced efficacy of immunotherapy in colorectal cancer patients with livermetastasis [61].

In the tissue samples analyzed in this study, the expression of PD-L1 in metastatic tumors was higher than that in primary tumors, similarly to the results published by Wei et al. Furthermore, the results regarding CD4 and CD8 cells were consistent with data published by other authors: in our samples, the expression of CD4 and CD8 was higher in metastatic tumors (both at the tumor margin and in the central region), when assessed as a percentage. Furthermore, the expression of these cells in the central region of the tumor, when measured as the number of cells per mm^2^, was also higher in metastatic tumors.

Ko et al. similarly evaluated the correlation between the density of immune cell infiltration in CRC tissue and the presence of TILs—specifically, CD3+ and CD8+ cells—and observed that a higher Immunoscore was significantly associated with favorable tumor behavior, including lower rates of vascular, lymphatic, and perineural invasion as well as reduced lymph node and distant metastases. Moreover, colorectal cancer tissues with high levels of CD8+ lymphocytes exhibited higher PD-L1 expression on both tumor and immune cells, when compared with tissues with low CD8+ lymphocyte infiltration [62]. In our study, patients exhibiting a low percentage of CD8+ cells in the center of the primary tumor showed improved survival outcomes compared those with higher CD8+ levels; however, no significant association was identified when CD8+ cell density was analyzed in terms of the number of cells per mm^2^, nor did we observe any association between the number of CD8+ cells in metastatic tissue and survival. These results are in contradiction with the meta-analysis conducted by Mei et al., who reported that high CD8+ cell infiltration in the tumor stroma was associated with prolonged overall survival, while CD3+, CD8+, and FoxP3+ cells located in the tumor center were not statistically relevant markers [63].

We also demonstrated a correlation between PD-L1 expression and overall survival (OS): among patients with PD-L1 expression <1% on tumor cells in the primary tumor, OS was significantly greater when compared with that of those with PD-L1 expression between 1 and 5%. In contrast, no significant impact of tumor cell PD-L1 expression on survival was observed in metastatic lesions. Similarly, PD-L1 expression on tumor-infiltrating immune cells showed no association with OS in either primary tumors or metastases. The connection between PD-L1 expression and the outcomes in our study is in accordance with the meta-analysis conducted by Wang et al., in which PD-L1 expression was found to be significantly correlated with lymphatic metastases, tumor size, differentiation, and vascular invasion. Moreover, PD-L1 expression could serve as an independent indicator of poor prognosis in colorectal cancer (CRC) [64].

Another important element of the microenvironment in our study is tumor-associated macrophages (TAMs) characterized by CD68 surface markers. TAMs can be broadly categorized into two phenotypic subtypes: M1 and M2. M1 macrophages, which are activated via the classical pathway, take part in promoting the anti-tumor immune response by driving Th1 lymphocyte activity; furthermore, they initiate tumor killing through the induction of a chronic inflammatory state in the tumor microenvironment via the secretion of pro-inflammatory cytokines (IL-12, TNF-α). In contrast, M2 macrophages, which are activated via the alternative pathway, act immuno suppressively, support tumor progression and evasion of immune surveillance, and may lead to tumor stroma remodeling and neoangiogenesis [65,66,67,68,69]. The TAM phenotype can be identified based on the expression of surface markers: CD68 is expressed on all macrophages, inducible NOS is characteristic of the M1 phenotype, and CD163 is a marker of M2 macrophages [70,71]. Moreover, in response to microenvironment stimulation, M2-polarized macrophages can switch to M1 macrophages and vice versa [72]. The univariate analysis revealed an association between the percentage of CD68+ cells in the center of the primary tumor and the total number of cells. Patients with low expression of CD68 in terms of percentage demonstrated better survival outcomes compared with those with higher CD68 levels; however, no such association was observed in the analysis based on the number of CD68+ cells per mm^2^.

We also analyzed the difference in expression of CD31 on endothelial cells—which is used to assess tumor angiogenesis and αSMA expression as a marker of smooth muscle cells and activated fibroblasts in the tumor stroma—between primary and metastatic tumors; however, no significant differences were found.

The metastatic microenvironment also influences the response to anticancer therapy. Kim et al. observed that brain metastases from lung cancer responded less effectively to anti-PD-1 antibodies than the primary tumor, most likely due to reduced infiltration of PD-1-positive T lymphocytes in the metastatic tissue [73]. Tumors with high infiltration of myeloid-derived suppressor cells (MDSCs), limited infiltration of CD4+ and CD8+ T cells, reduced programmed death receptor (PD) expression and decreased expression of class I and class II HLA molecules are categorized as microsatellite-stable (MSS) colorectal cancer (CRC), commonly referred to as “cold tumors.” This immunosuppressive tumor microenvironment in CRC results in a poor response to monotherapy with programmed death-1 (PD-1) or programmed death-ligand 1 (PD-L1) inhibitors; however, these tumors may be treated using chemotherapy [74,75].

PD-L1 expression in tumor cells has been validated as a predictive biomarker for response to anti-PD-1 and anti-PD-L1 immunotherapies in other malignancies [76,77,78]. Immune checkpoint therapy was granted regulatory authorization in 2017 for the treatment of heavily mutated tumors that are mismatch repair-deficient (dMMR) or have high levels of microsatellite instability (MSI-H). Patients with CRC exhibiting dMMR/MSI-H have shown increased sensitivity to anti-PD-1/PD-L1 antibody therapy and achieved more prolonged and durable responses, compared with those characterized by pMMR/MSI-L [79,80,81,82,83,84]. This observed difference in therapeutic response is most likely attributable to the distinct composition of the TME between pMMR and dMMR CRC, with pMMR tumors exhibiting a higher proportion of PD-positive TILs and increased PD-L1 expression on tumor cells compared with dMMR tumors [85,86].

The impact of TAM infiltration on the course of CRC and patient survival tends to vary depending on the study. According to a meta-analysis conducted by Li et al., a high density of the CD68+ TAM subset was correlated with improved 5-year OS, whereas neither the CD68+NOS2+ M1 subset nor the CD163+ M2 subset showed any correlation with the 5-year OS [87]. In contrast, negative impacts of high-density TAM infiltration on OS have been observed in other cancers, including breast [88], gastric, ovarian, bladder, and head and neck cancers [89]. In the cited studies, TAM density was defined in terms of the number of cells per mm^2^, which may explain the differences in results compared with our study. Assessing the density of immune cell infiltration as a percentage of all immune infiltrating cells appears to better reflect the character of tumor immune infiltration, when compared with evaluation based on the number of cells per mm^2^. Percentage-based assessment of lymphocyte infiltration provides insight into the proportion of immune cells within the tumor and enables evaluation of the relative contributions of immune, neoplastic, and stromal cell populations. This can correlate with the immunological status of the tumor (e.g., immunosuppressive vs. anti-neoplastic), rather than the number of cells per surface. It also enables more accurate comparison of samples, as the number of cells may vary depending on the extent of necrosis, technical processing, and the sampling site.

In pMMR colorectal cancer, a greater polarization of macrophages toward the M2 phenotype has been observed in contrast to dMMR, which is associated with a worse prognosis [90].

Immune microenvironment heterogeneity between different metastatic lesions within the same patient has also been evidenced, which may explain the diverse clinical behaviors of metastases following treatment. In their study of a patient with high-grade serous ovarian cancer after multiple lines of therapy, Jiménez-Sánchez et al. demonstrated that metastases which progressed were characterized by immune cell exclusion, whereas regressing and stable metastases were infiltrated by CD4+ and CD8+ T lymphocytes [91]. These results are in concordance with the “seed and soil” theory, in which “seeds” represent cancer cells, while the “soil” refers to the microenvironment in which metastases can successfully develop [92]. As the microenvironments of different organs vary and tumor–TME interactions shape the phenotype of both the tumor and its surrounding environment, it is expected that metastases arising from the same primary tumor in a single patient may differ depending on the organ in which they form [93,94,95,96].

Despite the observed increase in overall immune cell infiltration in metastatic lesions compared with primary tumors, the functional orientation of infiltrating cells is crucial for effective antitumor immunity. Metastatic niches—particularly in the liver—are often enriched in immunosuppressive cell populations such as M2-polarized tumor-associated macrophages, regulatory T cells, and myeloid-derived suppressor cells, which can counteract the cytotoxic activity of effector lymphocytes [97,98,99]. These immunosuppressive cells secrete inhibitory cytokines, including IL-10 and TGF-β, and express immune checkpoint ligands such as PD-L1, thereby promoting local immune evasion despite an increased immune cell density [100,101,102]. Furthermore, persistent antigen stimulation at metastatic sites can induce T cell exhaustion, characterized by impaired proliferation and reduced cytotoxic function [103]. In addition, the liver is a physiologically immunotolerant organ, promoting regulatory immune signaling pathways that can be exploited by colorectal cancer metastases to create an immunosuppressive microenvironment [104,105].

**Figure 6:**
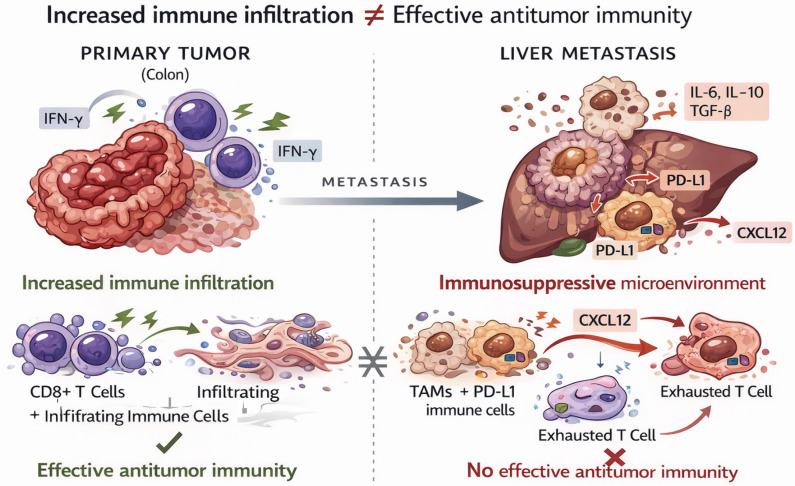
Immune infiltration and functional immunosuppression in metastatic colorectal cancer. Schematic illustration comparing the tumor microenvironment of primary colorectal cancer and liver metastases, highlighting that increased immune cell infiltration in metastatic lesions may coexist with PD-L1 expression, immunosuppressive signaling, as well as cytokines and chemokines (e.g., IL-6, IL-10, TGF-β, CXCL12) and T cell exhaustion, resulting in ineffective antitumor immunity. Created with the assistance of artificial intelligence tools. The authors take full responsibility for the scientific content, interpretation, and conclusions presented in the figure.

Analyses of colorectal cancer liver metastases have demonstrated that immune checkpoint–mediated signaling pathways, the accumulation of immunosuppressive myeloid cell populations, and extensive stromal remodeling collectively and decisively shape the metastatic niche, thereby constraining the development of effective antitumor immune responses (Figure 6) [106,107]. Moreover, large-scale immune profiling studies have consistently indicated that qualitative, functional, and spatial characteristics of the immune microenvironment provide substantially greater prognostic and predictive value with respect to clinical outcome and therapeutic response than immune cell density considered in isolation [108,109]. Notably, recent investigations in other gastrointestinal malignancies have further revealed that tumor microenvironmental features, when evaluated in conjunction with intrinsic tumor cell characteristics, can robustly predict metastatic potential, thus emphasizing the broader relevance of comprehensive, tissue-based assessment of the tumor microenvironment [110,111]. Consequently, increased immune infiltration observed within metastatic lesions should be interpreted with careful consideration of immune cell composition, spatial organization, and functional state, rather than being regarded solely as a quantitative measure.

These findings are concordant with our own observations and collectively underscore the importance of integrated analyses of matched primary tumors and corresponding metastatic lesions for elucidating mechanisms of immune dysfunction that emerge during metastatic progression.

A limitation of this study is the small size of patient subgroups, which may restrict the interpretability of subgroup analyses. The cohort size reflects the specific inclusion criteria of this retrospective study; however, all consecutive eligible patients treated at our institution were included, ensuring internal consistency and minimizing selection bias. The wide range of follow-up durations reflects heterogeneous clinical outcomes, with shorter follow-up mainly due to early disease progression or death.

The retrospective and exploratory design precluded the definition of a predefined primary endpoint and the performance of an a priori power calculation, and some analyses may therefore be underpowered. Power analysis showed low power for survival analyses stratified by neuro invasion in the primary tumor (68.82%), warranting cautious interpretation of negative or borderline results. In contrast, analyses based on CD8 and CD68 expression in the tumor center demonstrated acceptable power (82.78% and 82.09%, respectively), and the Wilcoxon signed-rank test assessing CD8+ cell differences between primary and metastatic lesions showed very high power (94.95%), supporting the robustness of this finding.

Although the number of cases did not allow for extensive subgroup analyses, including comparisons by gender, the study design—based on paired analyses of primary and metastatic lesions in the same patients—provides robust and biologically relevant insights into differences between tumor microenvironments. The inclusion of a single case of pancreatic metastases did not affect the results and was retained to maintain cohort completeness and methodological consistency. We believe that the methodological rigor, comprehensive tissue analysis, and full inclusion of all eligible cases in this study support the reliability and validity of the presented results. The absence of several clinical and pathological variables (including M stage, KRAS status, and tumor budding) was primarily due to the retrospective nature of the study and changes in routine diagnostic practice over time. In particular, extended molecular profiling (KRAS/NRAS/BRAF and MSI/dMMR) was not uniformly performed in earlier years as part of routine clinical care, with testing often limited to situations of direct therapeutic relevance. Nevertheless, the observed level of clinical benefit—as confirmed by the treatment responses—is noteworthy, especially in the context of previous clinical trials conducted in heavily pre-treated patients with metastatic colorectal cancer.

## 5. Conclusions

In this study, we comprehensively characterized the tumor microenvironment in both primary colorectal tumors and their corresponding metastases, including an evaluation of the tumor microvasculature. Multiple complementary analytical approaches were applied to obtain more detailed insights into the complex interactions between tumor cells and infiltrating immune and stromal components.

Our findings indicate that primary and metastatic tumors exhibit distinct microenvironmental profiles with respect to their immune cell composition and spatial distribution. Metastatic lesions were dominated by immune cell populations associated with an immunosuppressive phenotype, which may contribute to disease progression and therapeutic resistance. Furthermore, increased PD-L1 expression was observed in metastatic tumors, highlighting the dynamic nature of immune escape mechanisms during tumor evolution. These observations underscore the potential clinical relevance of incorporating tumor immune characteristics into therapeutic decision-making and post-treatment surveillance strategies and support further investigation of the tumor microenvironment as a target for personalized treatment approaches.

## Figures and Tables

**Table 1:** Antigens and corresponding immune cells.

Antigens	Typeof Immune Cells	References
CD4	CD4+ T cells	[33]
CD8	CD8+ T cells	[34,35]
CD15	Granulocytes (neutrophils)	[36]
CD56	NK lymphocytes	[37]
CD68	Macrophages	[38]
CD31	Tumor vessels	[39,40]
CD208	Dendritic cells	[41,42]
aSMA	Cancer-associated fibroblasts	[43]
PD-L1	Tumor cells, tumor-infiltrating immune cells	[44]

**Table 2:** Patient characteristics.

Feature	Number of Patients (*n*)	Percentage (%)
Sex		
Male	8	26.67
Female	22	73.33
Stage		
T1	0	0
T2	2	6.67
T3	27	90
T4	1	3.33
Nodes		
N0	10	33.33
N1a	8	26.67
N1b	3	10
N1c	3	10
N2a	4	13.33
N2b	2	6.67
Metastases		
M0	3	10
M1	5	16.67
M1A	2	6.67
M1B	1	3.33
M1C	0	0
Data on M stage not available	19	63.33
Grading		
G1	2	6.67
G2	27	90
G3	1	3.33
Angioinvasion		
No	13	43.33
Yes	14	46.67
Not known	3	10
Neuroinvasion		
No	16	53.33
Yes	11	36.67
Not known	3	10
KRAS Mutation		
No	3	10
Yes	3	10
Not known	24	80
Tumor Budding		
Low	2	6.67
Medium	1	3.33
High	1	3.33
Not known	26	86.67
Side of Colon		
Right	8	26.67
Left	22	73.33
Presence of Other Cancer		
No	26	86.67
Yes	4	13.33
Timing of Metastasis		
Synchronous tumors	21	70
Metachronous tumors	9	30
Presence of Obstruction		
No	18	60
Yes	8	26.67
Not known	4	13.33
Presence of Perforation		
No	24	80
Yes	3	10
Not known	3	10
Clear Margins in Histopathology		
No	0	0
Yes	30	100
Status Post-chemotherapy Prior to Material Collection		
No	9	30
Yes	17	56.67
Not known	4	13.33
Location of Metastases		
Liver	29	96.67
Pancreas	1	3.33
Other	0	0
Local Recurrence		
No	27	90
Yes	3	10
Number of Metastatic Lesions		
Single	11	36.67
Multiple	19	63.33

**Table 3:** (**a**) Median results obtained for the analyzed primary tumor and metastatic lesion specimens. (**b**) PD-L1 results obtained for the analyzed specimens.

**(a)**								
**Antigen Analyzed in the Specimen**								
	**INV [%]**		**INV/[mm^2^]**		**CENTR [%]**		**CENTR [mm^2^]**	
	**Primary**	**Metastatic**	**Primary**	**Metastatic**	**Primary**	**Metastatic**	**Primary**	**Metastatic**
CD4	14.95	32.3	513.67	1413.17	3.25	3.34	145	110.67
CD8	10.53	16.86	370.5	843.83	2.19	2.25	110	93.67
CD15	9.77	11.45	431.83	567.33	-	-	-	-
CD56	0.32	0.76	10.33	33.65	0.18	0.14	6	6
CD68	39.54	44.2	1773.67	2326.5	22.1	23.72	994.67	1289.17
CD31	-	-	47.33	70.33	-	-	38.83	39.33
αSMA	64.63	55.39	-	-	-	-	-	-
**(b)**								
**Antigen**			**Median**		**Minimum**	**Maximum**		
pPD-L1 (TC)			1		1	2		
pPD-L1 (IC) 1—low, 2—moderate, 3—high			2		1	3		
mPD-L1 (TC)			1		1	2		
mPD-L1 (IC) 1—low, 2—moderate, 3—high			3		2	3		

INV, invasive margin of the tumor; CENTR, tumor center; TC, tumor cells; IC, tumor-infiltrating immune cells; p, molecules analyzed in primary tumor specimens; m, molecules analyzed in the metastatic lesion specimens. Expression of PD-L1: low, <1%; moderate, 1–5%; high, >5%.

**Table 4:** Statistically significant correlations that occurred only in [%].

Correlations	R	*p*
pCD4 CENTR [%] and presence of KRAS mutation.	−0.905	0.013
pCD8 INV [%] and presence of perforation.	−0.414	0.032
pCD8 CENTR [%] and location of metastasis—liver or pancreas	0.699	<0.001
mCD8 CENTR [%] and presence of neuroinvasion	0.428	0.026
mCD15 INV [%] and time of metastasis appearance (synchronous/metachronous)	0.407	0.025
pCD15 INV [%] and number of metastatic lesions—single or multiple	−0.579	<0.001
paSMAINV [%] and presence of angioinvasion	−0.473	0.013
paSMAINV [%] and tumor budding	0.978	0.022
maSMAINV [%] and side of the colon (right/left)	−0.420	0.020
pCD56 CENTR [%] and number of metastatic lesions—single or multiple	−0.474	0.008
pCD68 CENTR [%] and feature M	0.607	0.048
pCD68 CENTR [%] and time of metastasis occurrence (synchronous/metachronous)	−0.372	0.043
pCD68 CENTR [%] and presence of neuroinvasion	0.491	0.009
pCD68 CENTR [%] and presence of KRAS mutation.	−0.832	0.040
pCD68 INV [%] and tumor budding	0.100	<0.001
mCD68 CENTR [%] and presence of angioinvasion	0.411	0.033
mCD68 CENTR [%] and presence of neuroinvasion	0.615	<0.001

R, correlation coefficient; *p*, statistical significance level; INV, invasive margin of the tumor; CENTR, tumor center; p, molecules analyzed in primary tumor specimens; m, molecules analyzed in metastatic lesion specimens; [%], percentage of cells analyzed in relation to the rest of the cells in a given area examined.

**Table 5:** Statistically significant correlations that occurred only in /mm^2^.

Correlations	R	*p*
pCD4 CENTR/mm^2^ and number of metastatic lesions—single or multiple	−0.5471	0.002
pCD8 CENTR/mm^2^ and location of metastasis—liver or pancreas	0.804	<0.001
mCD8 INV/mm^2^ and tumor budding	0.957	0.043
mCD8 INV/mm^2^ and tumor budding	0.957	0.043
mCD8 INV/mm^2^ and number of metastatic lesions—single or multiple	0.465	0.010
mCD8 CENTR/mm^2^ and presence of neuroinvasion	0.384	0.048
pCD15 INV mm^2^ and number of metastatic lesions—single or multiple	−0.557	0.001
pCD31 INV/mm^2^ and presence of other malignancies	−0.361	0.050
pCD31 INV/mm^2^ and presence of perforation	0.565	0.002
pCD31 CENTR/mm^2^ and presence of perforation	0.386	0.047
pCD56 CENTR/mm^2^ and number of metastatic lesions—single or multiple	−0.480	0.007
mCD56 INV/mm^2^ and previous chemotherapy	−0.382	0.037
pCD68 CENTR/mm^2^ and local recurrence	−0.404	0.027
pCD68 INV/mm^2^ and time to metastasis appearance (synchronous/metachronous)	0.408	0.025
mCD68 CENTR/mm^2^ and presence of other malignancies	0.436	0.016
mCD68 INV/mm^2^ and location of metastasis—liver or pancreas	−0.375	0.041
mCD68 INV/mm^2^ and number of metastatic lesions—single or multiple	0.502	0.005
pCD68 INV/mm^2^ and tumor budding	0.958	0.042
mCD68 CENTR/mm^2^ and presence of neuroinvasion	0.525	0.005
pPD-L1 (TC) mm^2^ and location of metastasis—liver or pancreas	0.371	0.043
mPD-L1 (TC) and side of the colon (right/left)	−0.480	0.007

R, correlation coefficient; *p*, statistical significance level; INV, invasive margin of the tumor; CENTR, tumor center; TC, tumor cells; p, molecules analyzed in primary tumor specimens; m, molecules analyzed in metastatic lesion specimens; /mm^2^, number of cells analyzed per mm^2^.

**Table 6:** Results of univariable Cox proportional hazard analysis.

	HR	95% CI	*p*
pCD8 CENTR [%]	1.193	1.030–1.380	0.018
pCD8 CENTR/mm^2^	1.003	1.0003–1.005	0.030
pCD68 CENTR [%]	1.040	1.002–1.088	0.039
pPD-L1 (TC)	5.835	2.074–16.419	<0.001
Presence of neuroinvasion	0.268	0.091–0.784	0.016

HR, hazard ratio; *p*, statistical significance level; CENTR, tumor center; TC, tumor cells; p, molecules analyzed in primary tumor specimens; [%], percentage of cells analyzed in relation to the rest of the cells in a given area examined; /mm^2^, number of cells analyzed per mm^2^.

**Table 7:** Results of multivariable Cox proportional hazard analysis.

	HR	95% CI	***p***
pPD-L1 (TC)	5.43	1.89–15.61	0.0017

HR, hazard ratio; *p*, statistical significance level; TC, tumor cells; p, molecules analyzed in primary tumor specimens.

**Table 8:** Wilcoxon signed-rank test comparing paired parameters of the primary (p) and metastatic (m) tumor microenvironments.

Pairs of Variables	Wilcoxon Signed-Rank Test			
	N of Significant	t	Z	*p*
pCD8 INV % and mCD8 INV [%]	30	61	3.53	0.0004
pCD8 INV/mm^2^ and mCD8 INV/mm^2^	30	62	3.507	0.0005
pPD-L1 (IC) 1 = low, 3 = high and mPD-L1 (IC) 1 = low, 3 = high	14	19.5	2.072	0.0383

N, number of pairs; t, test statistic; Z, standardized test statistic; *p*, statistical significance level; INV, invasive margin of the tumor; IC, tumor-infiltrating immune cells; p, molecules analyzed in primary tumor specimens; m, molecules analyzed in metastatic lesion specimens; [%], percentage of cells analyzed in relation to the rest of the cells in a given area examined; /mm^2^, number of cells analyzed per mm^2^. PD-L1 expression: <1%, low; 1–5%, moderate; >5%, high.

**Table 9:** Spearman’s rank-order correlation between PD-L1 expression and CD8+ T cell infiltration in metastatic lesions.

Pair of Variables	Spearman’s Rank-Order Correlation			
	N of Significant	R Spearman	t(N − 2)	*p*
mPD-L1(IC) 1—low, 3—high and mCD8 INV [%]	30	0.372	2.118	0.043
mPD-L1(IC) 1—low, 3—high and mCD8 INV/mm^2^	30	0.468	2.799	0.009

N, number of pairs analyzed; R Spearman, correlation coefficient; t(N − 2), *t*-test statistic; *p*, statistical significance level INV, invasive margin of the tumor; IC, tumor-infiltrating immune cells; m, molecules analyzed in metastatic lesion specimens; [%], percentage of cells analyzed in relation to the rest of the cells in a given area examined; /mm^2^, number of cells analyzed per mm^2^. PD-L1 expression: <1%, low; 1–5%, moderate; >5%, high.

**Table 10:** Mann–Whitney U-test comparing CD8+ T cell infiltration between metastatic lesions depending on PD-L1 expression on immune cells (mPD-L1 IC).

Variable	Mann–Whitney U Test (Adjusted for Continuity); Relative to a Variable: mPD-L1 (IC)—Group Moderate; Group High									
	Sum. Rang Group Moderate	Sum. Rang Group High	U	Z	*p*	Adjusted	*p*	N Valid Group Moderate	N Valid Group High	2 × 1 Side Accuracy *p*
mCD8 INV [%]	341	124	58	1.98	0.048	1.98	0.048	19	11	0.047
mCD8 INV/mm^2^	353	112	46	2.49	0.013	2.50	0.013	19	11	0.011

Sum. Rang Group moderate/Group high, sum of ranks for each group; U, Mann–Whitney U statistic; Z, standardized Z-score; Adjusted *p*, *p*-value corrected for continuity; N valid Group moderate/Group high, number of valid observations in each group; 2 × 1-side accuracy *p*, exact two-tailed *p*-value; *p*, statistical significance level; INV, invasive margin of the tumor; IC, tumor-infiltrating immune cells; m, molecules analyzed in metastatic lesion specimens; [%], percentage of cells analyzed in relation to the rest of the cells in a given area examined; /mm^2^, number of cells analyzed per mm^2^. PD-L1 expression: <1%, low; 1–5%, moderate; >5%, high.

## Data Availability

Data are contained within the article.

## Supplementary Materials

The following supporting information can be downloaded at: https://www.mdpi.com/article/10.3390/cancers18040566/s1.
