# Supplementary material for: Disparities in Tumor Microenvironment Between Primary and Metastatic Colorectal Cancer: Impact on Immune Infiltration and Survival
Source: Cancers (Basel). 2026 Feb 9;18(4):566. doi: 10.3390/cancers18040566 (PMC12938962; doi:10.3390/cancers18040566)
Supplement: Supplementary file 1 [file cancers-18-00566-s001.zip › cancers-4099038-supplementary.pdf]

	Catalog number	Antibodes
1	IR64961-2-PAT	FLEX Mono Mo a Hu CD4, cl 4B12, RTU
2	IR62361-2-PAT	FLEX Mono Mo a Hu CD8, cl C8/144B, RTU
3	IR62861-2-PAT	FLEX Mono Mo a Hu CD56, cl 123C3, RTU
4	IR60961-2-PAT	FLEX Mono Mo a Hu CD68, cl KP1, RTU
5	IR61061-2-PAT	FLX Mo aHu CD31 Endothel Cell JC70A, RTU
6	IR70061-2-PAT	FLX Moa Hu Muscle Actin HHF35 RTU Link
7	IR64261-2-PAT	FLEX Mono Mo a Hu CD138, cl MI15, RTU
8	IR06261-2-PAT	FLEX Mono MxH CD15, cl Carb-3 RTU
9	IR60461-2-PAT	FLEX Mono Mo a Hu CD20cy, cl L26, RTU
10	M365329-2-PAT	Mono Mo a Hu PD-L1, cl 22C3
